# Cholinergic and ghrelinergic receptors and KCNQ channels in the medial PFC regulate the expression of palatability

**DOI:** 10.3389/fnbeh.2015.00284

**Published:** 2015-10-26

**Authors:** Marc A. Parent, Linda M. Amarante, Kyra Swanson, Mark Laubach

**Affiliations:** ^1^The John B. Pierce LaboratoryNew Haven, CT, USA; ^2^Department of Neurobiology, Yale University School of MedicineNew Haven, CT, USA; ^3^Department of Biology and Center for Behavioral Neuroscience, American UniversityWashington, DC, USA

**Keywords:** KCNQ, muscarinic, ghrelin, reward, licking, prefrontal

## Abstract

The medial prefrontal cortex (mPFC) is a key brain region for the control of consummatory behavior. Neuronal activity in this area is modulated when rats initiate consummatory licking and reversible inactivations eliminate reward contrast effects and reduce a measure of palatability, the duration of licking bouts. Together, these data suggest the hypothesis that rhythmic neuronal activity in the mPFC is crucial for the control of consummatory behavior. The muscarinic cholinergic system is known to regulate membrane excitability and control low-frequency rhythmic activity in the mPFC. Muscarinic receptors (mAChRs) act through KCNQ (Kv7) potassium channels, which have recently been linked to the orexigenic peptide ghrelin. To understand if drugs that act on KCNQ channels within the mPFC have effects on consummatory behavior, we made infusions of several muscarinic drugs (scopolamine, oxotremorine, physostigmine), the KCNQ channel blocker XE-991, and ghrelin into the mPFC and evaluated their effects on consummatory behavior. A consistent finding across all drugs was an effect on the duration of licking bouts when animals consume solutions with a relatively high concentration of sucrose. The muscarinic antagonist scopolamine reduced bout durations, both systemically and intra-cortically. By contrast, the muscarinic agonist oxotremorine, the cholinesterase inhibitor physostigmine, the KCNQ channel blocker XE-991, and ghrelin all increased the durations of licking bouts when infused into the mPFC. Our findings suggest that cholinergic and ghrelinergic signaling in the mPFC, acting through KCNQ channels, regulates the expression of palatability.

## Introduction

Consummatory behavior modulates neuronal activity in the medial prefrontal cortex (mPFC) of rats and primates (Petykó et al., 2009, 2015; Bouret and Richmond, 2010; Horst and Laubach, 2012, 2013). For example, a recent study from our group (Horst and Laubach, 2013) found that population activity in the rostral prelimbic cortex was strongly modulated at the moment when rats initiated licking. These changes in spike activity were coterminous with 4–8 Hz phase locking in simultaneously recorded field potentials. It is possible that these signals are used to monitor the consequences of ongoing orolingual actions and are integrated with gustatory information, which has recently been shown to be encoded by neurons in the mPFC (Jezzini et al., 2013), to control reward-guided behaviors.

In a related recent study, we developed an operant incentive contrast task to study how rats learn to maximize consumption of rewarding solutions relative to less rewarding options (Parent et al., 2015). Pharmacological and optogenetic inactivations of the rostral prelimbic area shortened bouts of licking when rats consumed relatively high, but not low, levels of sucrose. Classic (Davis, 1973) and more recent investigations (e.g., Dwyer, 2012) have established that the duration of licking bouts in animals ingesting varying quantities of sucrose reflect the relative reward value (aka subjective value) of the solutions. Following inactivation of the mPFC, animals responded as if they were naive to the task. We interpreted these findings as evidence for the rostral mPFC being crucial for the expression of incentive contrast and for the deployment of learned feeding strategies.

In the incentive contrast task, performance depends on the animal’s ability to attend to changes in reward value (stop consumption when solution switches to the low value) and their motivation to consume (drive to consume a rewarding solution of high caloric content). Attention and motivation are both partially driven by the influences of acetylcholine (Voytko, 1996; Robbins, 2002; Chudasama et al., 2004; Bloem et al., 2014) and ghrelin (Kojima et al., 1999; Nakazato et al., 2001), respectively, in the brain. The mPFC has receptors for both of these neuromodulatory neurotransmitters (van der Zee and Luiten, 1999; Hou et al., 2006; Mani et al., 2014). Activation of muscarinic acetylcholine receptors (mAChRs) has been shown to change neuronal excitability (Brown and Passmore, 2009; Santini and Porter, 2010) via activation of the G_q_/G_11_-PLC-linked intracellular cascades (Suh and Hille, 2002; Zhang et al., 2003; Delmas and Brown, 2005). This cascade ultimately increases the excitability of neurons via closure of Kv7 (KCNQ) potassium channels and inhibition of the M-current. Modulation of M-currents in mPFC specifically has been shown to regulate mPFC dependent behaviors, such as in fear conditioning tasks (Santini and Porter, 2010). Increases in neuronal excitability by mAChRs may increase the influence of synaptic input to this region and provide an efficient mechanism for engagement of mPFC during arousal and attention. To our knowledge, no group has examined the role of mACh receptors or KCNQ channels in the control of consummatory behavior by the mPFC.

The orexigenic peptide ghrelin has recently been shown to enhance the excitability of dopamine neurons in the substantia nigra pars compacta (Shi et al., 2013). The G-protein-mediated activation of the intracellular pathway responsible for modulation of KCNQ channels by mAChR overlaps with ghrelinergic modulation of excitability. Activation of the ghrelin receptor—growth hormone secretagogue receptor (GHS-R)—triggers activation of the same intracellular pathway, and ultimate closure of KCNQ channels (Shi et al., 2013), as mAChRs. The published functional impact of mPFC M-current manipulation on excitability and behavior, together with the potential co-regulation of these same effector KCNQ channels by mAChR and GHS-R, suggests that consumption in a task that is dependent on mPFC may be regulated by both of these neurotransmitter systems. As with the muscarinic system described above, no group has examined the role of ghrelin receptors in the mPFC with regard to the control of consummatory behavior.

Here, we demonstrate that both systemic and mPFC infusions of the muscarinic receptor antagonist scopolamine decreased the duration of licking bouts during access to high value sucrose solutions when provided alternating access to high and low value solutions. These results are similar to what has been previously reported following reversible inactivation of mPFC (Parent et al., 2015), and suggest that blocking mACh receptors with scopolamine disrupts the same elements of neuronal processing that is similarly affected by total cortical inactivation via muscimol. Exactly the opposite result was obtained when cholinergic tone was enhanced locally in mPFC with infusion of the cholinesterase inhibitor physostigmine (aka eserine), activation of mAChR with the mAChR agonist oxotremorine, and blocking KCNQ channels linked to mAChR receptors with XE-991. Furthermore, infusion of ghrelin, which acts on the same KCNQ channels as the muscarinic system enhanced the same measure of palatability (bout duration) only when the high value sucrose solution was available. All four of these manipulations effectively block KCNQ channels and increases neuronal excitability (at least in brain slices, Guan et al., 2011; Pafundo et al., 2013). These stimulatory manipulations all selectively increased the duration of licking bouts when a high value sucrose reward was available, and had no impact on licking for a lower value solution. As the duration of licking bouts is thought to reflect the palatability (or hedonic value) of ingested fluids (Davis, 1973), the present study is the first to implicate cholinergic and ghrelinergic signaling in the mPFC, acting through KCNQ channels, in the expression of palatability.

## Materials and Methods

All procedures carried out in this set of experiments were approved by the Animal Use and Care Committee of the John B. Pierce Laboratory and conform to the guidelines set forth for the Ethical Treatment of Animals (National Institutes of Health).

### Animals

Twenty-five Long-Evans rats of 350–450 grams were used in this study. Animals were housed individually and kept on a 12/12 h light/dark cycle switching at 7:00 AM and 7:00 PM. Upon arrival, animals were given 1 week of habituation to their new environment with free access to rat chow followed by daily handling for 1 week. After habituation and initial daily handling, animals had regulated access to food to maintain their body weights at approximately 90% of their free-access weights. Rats typically received 14–18 g of food each day around 5 pm and were weighed daily throughout the period of training and testing in the incentive contrast licking task. Animals had free access to water throughout the experiments. Of the rats used in this study, three rats were removed either due to improper surgical placement of cannulas or drastic changes in behaviors following central infusions that permanently altered baseline behavioral performance following multiple drug infusions.

### Behavioral Apparatus

All animals were trained in sound-attenuating behavioral boxes (ENV-008; Med Associates) containing a single horizontally placed spout located on one wall at 6.5 cm from the floor and a house light at the top of the box. Control of pumps and behavioral quantification was done using a MedPC system version IV (Med Associates). The licking spout was custom built to allow the convergence of two independent solution lines stemming from two independent pumps at a single point (John B. Pierce Laboratory Instruments Shop). Licking was tracked optically as breakage of an infrared beam by the tongue between a custom built emitter/detector placed directly in front of the licking spout (John B. Pierce Laboratory Instruments Shop). Movement of the animal during licking was restricted via placement of two walls on either side of the spout. Solution lines were connected to 60 ml syringes and solution was made available to animals by lick-triggered, single speed pumps (PHM-100; Med Associates) which drove syringe plungers. Each lick activated a pump which delivered roughly 0.029 ml of fluid per pump activation, or an average of 9.7 microliters of fluid per lick.

### Behavioral Task

The incentive contrast licking task used in these experiments is the same as described previously (Parent et al., 2015). Briefly, animals were placed into the operant chamber for 30 min and had constant access to the spout. Two independent pumps delivered sucrose solution to the same spout and were loaded with syringes containing either high value 20% sucrose solution (wt/vol) or low value 4% sucrose solution (wt/vol). After animals were placed into the behavioral box, the MedPC script was started causing the house light to turn on. Licking at the spout initiated a 30-s epoch of access to the high value solution. Each lick was recorded and a lick occurring after the end of the 30-s epoch triggered a 30-s epoch of access of low value sucrose. These epochs of access continually switched back and forth between pumps and provided alternating access to high and low value solutions. At the end of the 30 min session, the house light turned off and animals stopped receiving sucrose solution. Quantification of behavior was implemented via analysis of both licking counts and metrics of licking microstructure such as duration of licking bouts, number of licking bouts, and intra-bout licking rates.

### Behavioral Data Analysis

Analysis of licking was carried out via custom scripts written in MATLAB. Detection and quantification of licking bouts were done as in previous studies (Gutierrez et al., 2010; Horst and Laubach, 2013; Parent et al., 2015). Specifically, bouts were defined as having at least three licks within 300 ms and with an inter-bout interval of 0.5 s or longer. The first 10 epochs during each behavioral session were used to analyze licking microstructure. Statistical analyses were performed using R^1^.

### Surgery

Prior to cannulation, animals were given 2–3 days of free access to rat chow and water. Animals were initially anesthetized using 3.5% isoflurane gas followed by intraperitoneal injections of ketamine and xylazine. The scalp was shaved clean and animals were injected with a bolus of carprofen subcutaneously. Animals were placed into a stereotaxic frame using non-penetrating ear bars and the skull was covered with iodine for 1 min. Iodine was wiped clean from the scalp and the eyes were covered with ophthalmic ointment to prevent drying over the span of the surgery. Lidocaine (0.3 ml) was injected under the scalp and an incision was made longitudinally along the skull. The skin was retracted laterally and all tissue was cleaned from the surface of the scalp. The skull was leveled by adjusting the stereotaxic apparatus to ensure bregma and lamda were within the same horizontal plane. Four screw were placed in the parietal skull plates for support of the guide cannulas. Craniotomies were drilled bilaterally in the frontal skull plates over the medial prefrontal cortex and 26 gauge guide cannulas with dummy cannulas were inserted into the medial prefrontal cortex at 1 mm dorsal to the target coordinate (AP: +3.6; ML: ±1.4 @ 12° from the midsagittal plane; DV: −4.0). Later, 33 gauge injection cannulas were used which extended 1 mm past the tip of the guide cannulas. Craniotomies were sealed and implants initially secured with cyanoacrylate and accelerator. The entire intra-cranial implants were then secured to the skull crews and covered with methyl methacrylate dental cement. Skin surrounding the implant was cleaned and maintained taut via placement of a metal suture placed posteriorly to the implant. The wound was covered in antibiotic ointment and rats were injected with intraperitoneally the antibiotic enrofloxacin.

Following surgery, once animals were able to maintain an upright posture and move around the recovery cage, the animals were placed back into the animal housing facility and were provided water containing the enrofloxacin antibiotic as well as carprofen for pain management for 2 days. Full access to food was provided. Animals were checked and weighed daily for 1 week following surgery. To prevent the removal of dummy cannulas during grooming, Kwik Cast silicon sealant was placed over the dummy cannula caps and removed when access to the cannulas was needed. After 1 week, animals’ body weights returned to presurgical levels, restricted access to rat chow was reinstated, and animals continued with daily behavioral testing sessions.

### Drug Infusions

Following recovery from surgery and a period of retraining in the task with restricted food access, a series of controls were performed on all rats. First, animals were exposed to the same duration and levels of isoflurane gas used during infusion of drug on test day as an initial gas control session. Second, a PBS control was carried out where the same volume of vehicle without drug was injected intraperitoneally or infused into the mPFC while the animals were anesthetized under isoflurane gas. Finally, on test day, animals were anesthetized via isoflurane gas and drug was injected intraperitoneally or infused centrally into the mPFC. Following test day, recovery sessions were carried out. Each rat received between 1 and 4 sessions of drug infusions during the time of this study, and took on average 2.6591 (*SD* = 1.8165) sessions to recover back to a baseline level of performance on the task.

Drugs used in this study included scopolamine, physostigmine, oxotremorine, XE-991, and ghrelin. All drugs were obtained from Tocris and made into solutions using sterile PBS with pH 7.4. Doses were based on published studies: systemic scopolamine—Sánchez-Resendis et al. (2012); intracortical scopolamine—Santini et al. (2012); physostigmine—Herremans et al. (1997); oxotremorine—Desai and Walcott (2006); XE-991—Santini and Porter (2010); ghrelin—Naleid et al. (2005).

### Confirmation of Cannula Placement

At the termination of experiments, animals were initially anesthetized with isoflurane gas and injected intraperitoneally with Euthasol. Animals were transcardially perfused first with 200 ml of cold saline solution followed by 200 ml of cold 4% paraformaldehyde. Brains were removed and post-fixed in a mixture containing 4% paraformaldehyde, 20% sucrose, and 20% glycerol. Brains were then cut into 100 μm-thick coronal slices using a freezing microtome. Brain sections were mounted onto gelatin-coated slides and Nissl stained via treatment with thionin. Thionin-treated slices were dried through a series of alcohol steps and cleared with Xylene. Slides were covered with permount and coverslipped. Sections were imaged using a Tritech Research scope (BX-51-F), Moticam Pro 282B camera, and Motic Images Plus 2.0 software. The most ventral point of the injection bolus was compared against the Paxinos and Watson atlas to confirm coordinates.

## Results

### Systemic Effects of the Muscarinic Antagonist Scopolamine

Scopolamine was administered systemically over a range of doses (PBS, 0.1 mg/kg, 0.3 mg/kg, 1.0 mg/kg) with intraperitoneal (IP) injections. Independent, one-way repeated measures analysis of variance (ANOVAs) were performed between control and drug administration sessions on epochs of access to high and low value sucrose. ANOVAs were carried out on descriptors of licking microstructure (e.g., mean duration of licking bouts, mean number of bouts) and mean lick counts across all 30-s epochs within a daily 30 min session. Global metrics of consummatory behavior were also tested with ANOVAs including total licks within a daily session and time spent engaged in the task prior to satiation. Effects of drugs were compared across sessions to avoid potential confounding factor of satiety.

**Figure 1:**
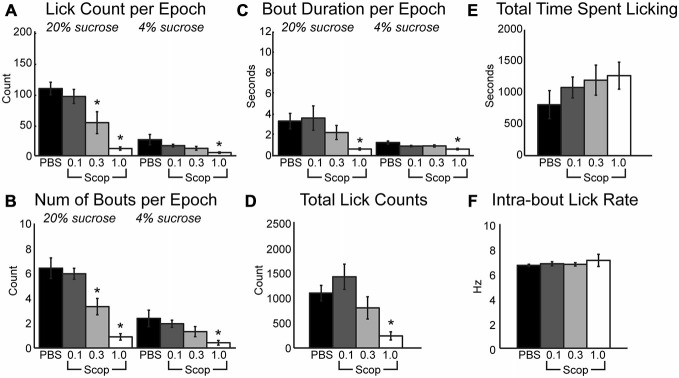
**Systemic scopolamine injections reduce performance on consummatory contrast task.** There is a dose-dependent decrease in consumption with systemic scopolamine injections. **(A)** Reduction in mean number of licks per 30 sepoch of access to high or low value sucrose solution across all epochs in a daily session at 0.3 mg/kg and 1.0 mg/kg systemic scopolamine injection relative to PBS vector. **(B)** There was a decrease in mean number of licking bouts per 30 sepoch at 0.3 mg/kg and 1.0 mg/kg systemic scopolamine. **(C)** Mean duration of licking bouts in epochs decreases at 1.0 mg/kg scopolamine. **(D)** Total number of licks across both high and low reward epochs combined in daily sessions were reduced at the 1.0 mg/kg dose of scopolamine. **(E)** There was a dose-dependent increase in the time spent by rats engaging in the task under injections of scopolamine. **(F)** Systemic injections of scopolamine did not alter the intra-bout licking rate, regardless of the given dose. *p < 0.05.

Clear effects of systemic scopolamine were apparent across the range of doses that were tested. During the 30-s epochs with access to either high or low levels of liquid sucrose, there was a significant decrease in mean licks per epoch (Figure 1A; HVS: [*F*_(3, 24)_ = 17.21, *p* < 0.001], LVS: [*F*_3,24_] = 5.84, *p* < 0.01), number of bouts per epoch (Figure 1B; HVS: [*F*_(3, 24)_ = 19.18, *p* < 0.001], LVS: [*F*_3,24_] = 4.02, *p* < 0.05), and duration of licking bouts (Figure 1C; HVS: [*F*_(3, 24)_ = 6.37, *p* < 0.01], LVS: [*F*_3,24_] = 5.77, *p* < 0.01) with increasing doses of scopolamine. There was also a significant decrease in the total number of licks (Figure 1D; [*F*_(3, 24)_ = 7.04, *p* < 0.01]) and a slight, yet insignificant, increase in the duration of time required to reach satiety within a session (Figure 1E; [*F*_(3, 24)_ = 0.91, *p* = 0.44]). *Post hoc* Tukey tests between PBS and the three drug levels found a significant change in licking, specifically with the mean number of licks and number of bouts during access to the high value sucrose solution, began to occur at the 0.3 mg/kg dose (*p* < 0.05). The 1 mg/kg dose strongly affected consumption of both the high and low reward solutions (*p* < 0.05 for all measures shown in Figures 1A–D). These reductions in consummatory behavior, especially at the higher dose of scopolamine, were independent of any effects on sensorimotor abilities, as there were no significant changes in the intra-bout licking rate at any dose injected (Figure 1F).

### Prefrontal Effects of the Muscarinic Antagonist Scopolamine

**Figure 2:**
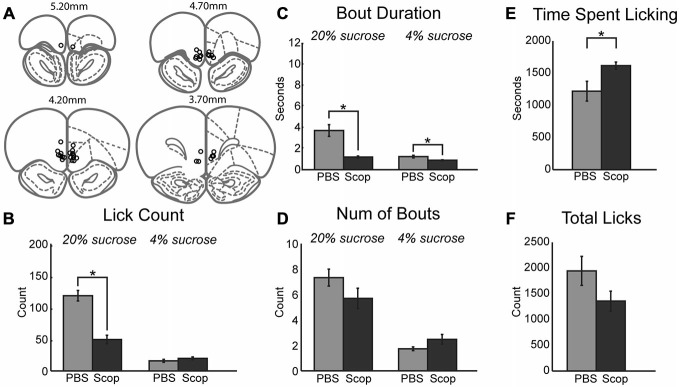
**Central infusions of scopolamine into mPFC reduce performance on consummatory contrast task. (A)** Central infusions of drugs across all rats were targeted to the medial prefrontal cortex. **(B)** There was a dramatic decrease in mean number of licks across epochs of access to the high value sucrose solution. **(C)** Scopolamine decreased the duration of licking bouts during access to both low and high value sucrose solutions. **(D)** There was a trending decrease and increase in the number of bouts performed within epochs of access to high and low value sucrose solutions, respectively. **(E)** Animals spent significantly more time engaged in the task following central infusions of scopolamine. **(F)** Scopolamine infusions led to a trending decrease in the total lick counts during daily sessions. *p < 0.05.

Having established systemic effects of scopolamine in the incentive contrast licking task, we next examined effects of local infusions of scopolamine within the mPFC. We focused on the same rostral region that contains licking-entrained neuronal activity (Horst and Laubach, 2013) and leads to the loss of incentive contrast effects and temporally fragmented licking when inactivated with muscimol (Parent et al., 2015). Figure 2A depicts cannula locations for all rats across all drug infusions into mPFC. Infusion of scopolamine (10 μg in 1 μl) resulted in a decrease in mean licks per epoch (Figure 2B; [*F*_(1, 6)_ = 18.68, *p* < 0.01]) and duration of licking bouts (Figure 2C; [*F*_(1, 6)_ = 39.18, *p* < 0.001]) during access to the high value solution. The effects on other measures were much less dramatic in comparison to the systemic data described above. While there was a decrease in the mean number of bouts initiated during access to the high value solution following infusion of scopolamine, this decrease did not reach significance (Figure 2D). During epochs of access to the low value solution there was a strong trend of increasing number of bouts (Figure 2D; [*F*_(1, 6)_ = 4.96, *p* = 0.068]) that were found to be of significantly shorter duration (Figure 2C; [*F*_(1, 6)_ = 22.04, *p* < 0.01). Overall, there was a significant increase in the length of time spent engaged in the task before reaching satiety (Figure 2E; [*F*_(1, 6)_ = 6.47, *p* < 0.05]), and a marginal decrease in licking throughout the entire session (Figure 2F; [*F*_(1, 6)_ = 2.61, *p* = 0.16]). While fluid intake for each session was recorded for each drug treatment in this study, only central infusions of scopolamine produced a significant change in volume consumed throughout the session, as measured by the average volume of fluid consumed per high value sucrose epoch divided by the average volume consumed for low value epochs in a given session (paired *t*-test: [*t*_(6)_ = 3.6669, *p* < 0.05]). For central infusions of scopolamine, high value sucrose intake decreased while low value sucrose intake increased.

### Prefrontal Effects of Physostigmine and Oxotremorine

**Figure 3:**
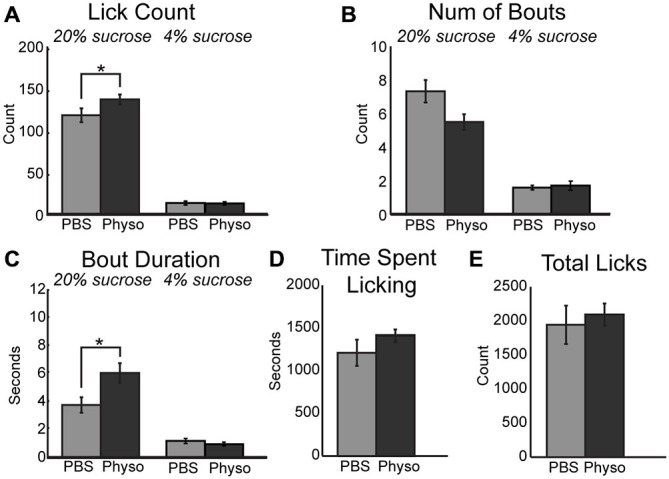
**Augmenting cholinergic tone in mPFC with physostigmine enhances consummatory behavior. (A)** Local infusion of physostigmine significantly enhanced the mean number of licks during epochs of access to the high value sucrose solution. **(B)** Physostigmine infusions led to a decreasing trend in the mean number of bouts during access to the high value sucrose solution. **(C)** Physostigmine enhanced consumption by significantly increasing the mean duration of licking bouts during access to the high value solution. **(D)** There was a slight but insignificant increase in the total time spent engaged in the task per 30 min session following infusion of physostigmine. **(E)** There was no significant difference with physostigmine infusions in the total number of licks emitted across the entire session. *p < 0.05.

If blockade of cholinergic signaling decreases consumption by reducing the ability of mPFC to contribute to the regulation of motivated behavior, it may be possible to augment the ability of rats to optimally negotiate the task via the upregulation of cholinergic tone locally within mPFC. This hypothesis was tested via the infusion of physostigmine, a classic cholinesterase inhibitor. Inhibition of acetylcholinesterase blocks the degradation of acetylcholine and generally increases cholinergic tone non-specifically regarding cholinergic receptor subtypes. Infusion of 10 μm physostigmine into mPFC augmented behaviors related to consumption and palatability during access to the high value sucrose. There was a significant increase in the mean number of licks per 30-s epoch (Figure 3A; [*F*_(1, 6)_ = 8.57, *p* < 0.05]). While there was only a trend toward a decrease in the number of bouts for the high value sucrose (Figure 3B; [*F*_(1, 6)_ = 4.66, *p* = 0.075]), there was a significant increase in the duration of licking bouts during sessions with physostigmine infusions (Figure 3C; [*F*_(1, 6)_ = 7.89, *p* < 0.05]). There was also a trending increase in the time spent licking during the session before reaching satiety (Figure 3D; [*F*_(1, 6)_ = 3.04, *p* = 0.132]). Physostigmine infusions did not alter the total number of licks emitted during the session (Figure 3E). Licking microstructure for the low value sucrose remained unchanged during physostigmine infusion sessions.

**Figure 4:**
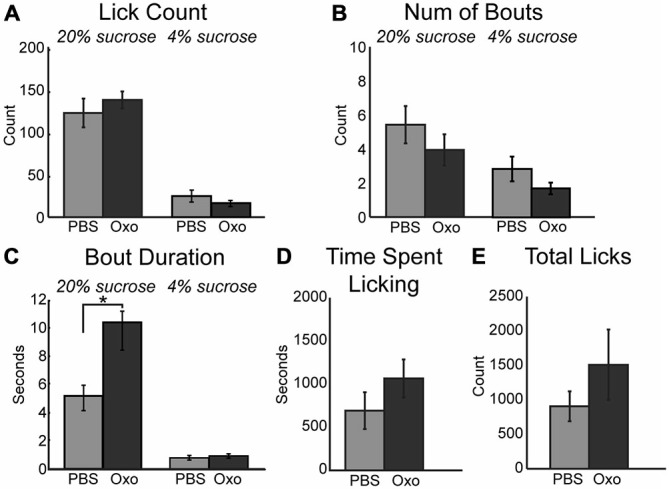
**Augmenting cholinergic tone in mPFC with oxotremorine enhances consummatory behavior. (A)** Local infusion of oxotremorine did not significant alter the mean number of licks emitted for the high value sucrose solution. **(B)** There was a decreasing trend in the number of bouts emitted for the high value sucrose solution following infusion of oxotremorine. **(C)** Oxotremorine significantly increased the duration of licking bouts for the high value sucrose solution. **(D)** Oxotremorine infusions did not significantly alter the time spent engaged in the task. **(E)** Infusions of oxotremorine did not significantly change the total number of licks emitted throughout the session. *p < 0.05.

The impact of scopolamine on consummatory behavior suggested that the increased consumption during the task following infusion of physostigmine may be rooted in modulation of muscarinic receptors. To explore this hypothesis, we infused a non-specific muscarinic receptor agonist oxotremorine into mPFC. While the mean number of licks per epoch remained unchanged during sessions with 10 μm oxotremorine infusions (Figure 4A), the total number of licks occurring during the session for the high value sucrose greatly increased ([*F*_(1, 4)_ = 8.04, *p* < 0.05]). Similar to the effects of physostigmine, infusion of oxotremorine showed a trend toward a decrease in the number of bouts for the high value sucrose solution (Figure 4B; [*F*_(1, 6)_ = 7.47, *p* = 0.052]). Infusion of oxotremorine significantly increased the duration of licking bouts for the high value sucrose solution (Figure 4C; [*F*_(1, 4)_ = 13.28, *p* < 0.05]). There was no significant change in the time spent engaged in the task (Figure 4D), nor was there a significant effect on total licks emitted across the entire session with oxotremorine infusions (Figure 4E).

### Prefrontal Effects of the KCNQ Channel Blocker XE-991

**Figure 5:**
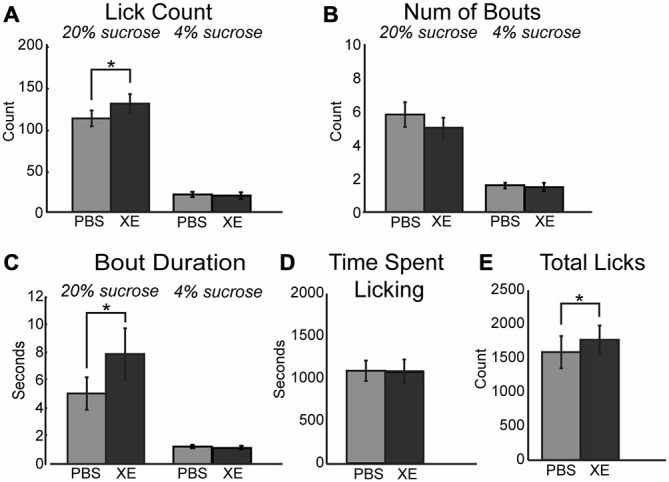
**Modulation of KCNQ channels in mPFC increased consumption for the high value sucrose solution. (A)** XE-991 significantly altered the mean number of licks occurring during epochs of access to the high value sucrose. XE-991 enhanced consumption of the high value sucrose solution. **(B)** Infusions of XE-991 did not significantly change the mean number of bouts for the high value sucrose solution. **(C)** XE-991 significantly increased the duration of bouts during access to the high value solution. **(D)** There was no impact on the time spent licking throughout the session follow infusions of XE-991. **(E)** There was a significant increase in total licks emitted with XE-991 infusions. *p < 0.05.

A critical downstream effector of muscarinic receptor activation within neurons is the KCNQ (Kv7.1) type potassium channel (Delmas and Brown, 2005; Brown and Passmore, 2009). Activation of these potassium channels decrease neuronal activity and promote neuronal synchrony in populations of neurons (e.g., in mPFC: Pafundo et al., 2013). Binding of acetylcholine to muscarinic receptors ultimately drives closure of KCNQ channels. This action drives neuron depolarization and increased neuronal excitability. Given the link between muscarinic receptors and KCNQ channels, alteration of KCNQ channel tone via direct pharmacological manipulations should alter consumption within our task. To test this hypothesis, 10 μM XE-991, a specific KCNQ channel blocker, was infused into mPFC. XE-991 significantly increased consumption during access to the high value sucrose solution via an increase in mean lick count (Figure 5A; [*F*_(1, 12)_ = 17.42, *p* < 0.01]). While there was no significant difference in the number of bouts emitted (Figure 5B), there was a significant increase in mean bout duration (Figure 5C; [*F*_(1, 12)_ = 13.81, *p* < 0.01]). There was no change in time spent licking during sessions with infusions of XE-991 (Figure 5D). Infusions of XE-991 did, however, have a significant increase in the total number of licks emitted throughout the behavioral session (Figure 5E; [*F*_(1, 12)_ = 6.202, *p* < 0.05]).

### Prefrontal Effects of Ghrelin

**Figure 6:**
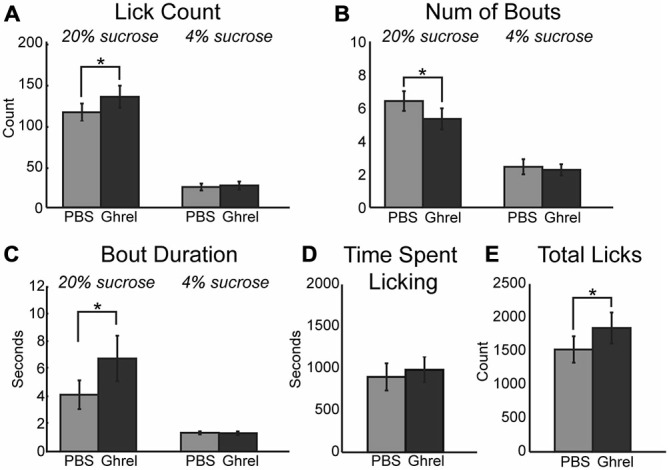
**Central infusions of ghrelin enhance consummatory behavior.** Infusion of ghrelin into mPFC increases consumption for the high value sucrose solution. Consumption of the low value sucrose solution remained unchanged. **(A)** Ghrelin significantly increased the mean number of licks during epochs of access to the high value sucrose solution. **(B)** There was a significant decrease in the number of bouts emitted during access to the high value sucrose solution following infusion of ghrelin. **(C)** Ghrelin infusions increased the duration of licking bouts during access to the high value solution. **(D)** Ghrelin had no effect on time spent engaged in the task. **(E)** Infusions of ghrelin increased the total number of licks emitted during the task. *p < 0.05.

Ghrelinergic modulation of intrinsic excitability in neurons is mediated via the same intracellular signaling pathway as the muscarinic modulatory system, specifically KCNQ channels (Li et al., 2013). Due to the presence of ghrelinergic receptors within the mPFC (Zigman et al., 2006) and the influence of muscarinic receptors on consumption reported above, ghrelin was infused centrally into the mPFC and its influence on behavior using the consummatory contrast task was tested. Similar to increased cholinergic tone, muscarinic receptor activation, and KCNQ channel inhibition, infusion of 1 μM ghrelin into the mPFC increased consumption of the high value sucrose solution via an increase in the mean number of licks (Figure 6A; [*F*_(1, 8)_ = 24.61, *p* < 0.01]) and total number of licks across the session (Figure 6E; [*F*_(1, 8)_ = 14.60, *p* < 0.01]). On average there were significantly fewer bouts for the high value sucrose solution (Figure 6B; [*F*_(1, 8)_ = 7.85, *p* < 0.05]). There was a marginal effect of increased mean duration of licking bouts for the high value sucrose solution (Figure 6C; [*F*_(1, 8)_ = 4.58, *p* = 0.065]). Similar to infusion of oxotremorine and XE-991, there was no effect of ghrelin on consumption during epochs of access to the low value sucrose solution, nor was there a change in the time spent engaged in the task (Figure 6D).

## Discussion

### Summary and Interpretation of Findings in the Present Study

In the present study, we found that decreasing cholinergic tone at muscarinic receptors with scopolamine both systematically and locally within the mPFC paralleled the results found following inactivation of mPFC using muscimol in an incentive contrast licking task (Parent et al., 2015). Decreased muscarinic tone in mPFC impairs performance on the task by decreasing the duration of licking bouts yielding a decreased rate of consumption. Further, we found that augmenting cholinergic tone locally within mPFC using physostigmine as well as more specifically via direct application of the muscarinic receptor agonist oxotremorine yielded an increase in task performance with greater consumption of the high value reward. A major downstream effector of muscarinic receptor activation is KCNQ (Kv7.1) potassium channel. Binding of acetylcholine to muscarinic receptors drives KCNQ channels into a closed conformation yielding neuronal depolarization and increased excitability. Blocking KCNQ channels with XE-991 drove an increase in task performance that paralleled what occurred following enhancement of cholinergic tone using physostigmine and oxotremorine. Finally, as the orexigenic peptide ghrelin has recently been shown to act on the same KCNQ channels (Shi et al., 2013), we evaluated its actions within the mPFC in some of the same animals, and found similar behavioral effects to the drugs that enhanced cholinergic tone and blocked KCNQ channels.

In all cases, the behavioral effects of the drugs were selective to the relatively higher concentration of sucrose that was tested (20%) and altered the same microstructural measure of licking, bout duration. Previous studies have found that the bout duration increases in proportion the concentration of sucrose (or other sapid nutrients) in the ingested solutions (Davis, 1973). Bout duration has been thus considered to reflect how palatable the solutions are to the animal (e.g., Davis and Perez, 1993) and reflect the relative reward value of a given solution (Grigson et al., 1993). Therefore, we conclude that cholinergic and ghrelinergic receptors and KCNQ channels in the medial PFC regulate the expression of palatability.

Our interpretation uses the phrase “expression of palatability” and not palatability *per se*. This is to emphasize the “readout” side of the control of consummatory behavior, and not the encoding of taste information or relative reward value, which has been proposed for other brain areas (agranular insular cortex and basolateral amygdala) and can be assayed using different behavioral measures, such as orofacial reactions (Grill and Norgren, 1978). The temporal control of consummatory behavior involves regulation of sensorimotor and autonomic/visceral systems. Sensorimotor control of consumption is regulated by a part of the medial agranular cortex (Yoshida et al., 2009) that is immediately adjacent to the mPFC area (rostral prelimbic cortex) that was the focus of the present study. Autonomic and visceral controls have been more traditionally emphasized for the prelimbic area (and the adjacent infralimbic cortex) (Terreberry and Neafsey, 1983) through its connections with the hypothalamus and autonomic midbrain and brainstem, as reviewed below. For example, the mPFC area that was studied here was recently shown to be involved in the regulation of breathing (Hassan et al., 2013). The rostral mPFC is well placed to coordinate the sensorimotor and autonomic motor systems through its descending projections (see Gabbott et al., 2005 for review).

### Potential Neuronal Mechanism of Cholinergic and Ghrelinergic Regulation of Palatability

The drugs that we tested might have altered the animals’ bout durations due to effects of the drugs on the ability of the mPFC to emit theta-range rhythms that normally accompany the initiation of consummatory behavior in rodents. Several recent studies have reported that neurons in the mPFC exhibit changes in firing rate around the initiation of licking (Petykó et al., 2009, 2015; Horst and Laubach, 2013). One of these studies (Horst and Laubach, 2013) also reported phasic changes in field potentials occur when rats initiate and terminate bouts of licks. The fields showed enhanced phase locking near the licking rhythm, between 6 and 8 Hz, a frequency range that is normally associated with “theta” in rodents. This rhythm might reflect a temporal synchronization of network-level activity that could serve to monitor the outcome of licking (Gutierrez et al., 2006) or could reflect a transient encoding of reward expectancy (van Wingerden et al., 2010), as proposed for similar signals in the orbitofrontal cortex.

Theta can be generated in several ways in the frontal cortex, by hippocampal inputs (which do not synchronize with licking: Vanderwolf, 1969), thalamocortical inputs (Hughes and Crunelli, 2007), and NMDA receptor-mediated spiking by layer 5 pyramidal neurons that are coupled to theta bursts by layer 2/3 pyramidal cells (Carracedo et al., 2013). These rhythms are enhanced by cholinergic agonists that act on the M current (Marrion, 1997), generated by KCNQ channels (Delmas and Brown, 2005; Brown and Passmore, 2009). *In vitro* slice physiology has shown that the application of the selective KCNQ channel blocker, XE-991, increases neuronal excitability, especially in response to low frequency inputs (<10 Hz; Guan et al., 2011; see also Pafundo et al., 2013 for effects in prefrontal cortical slices). Theta activity can be generated intracortically by regular spiking neurons in layer V (Carracedo et al., 2013). These cells are temporally gated by coterminous lower-frequency delta rhythms generated by intrinsic bursting cells (Carracedo et al., 2013). A disruption of the precise temporal interactions between these neurons, by any drug that acts on KCNQ channels or alters extracellular transmitters that act to regulate these channels, would thus disrupt the normal control of rhythmic behaviors that depend on neuronal processing within the cortical area of interest and/or within a collection of brain areas that control consummatory behavior in a coordinated manner.

We must point out that the interpretation of our findings are based on *in vitro* slice physiology studies, and not *in vivo* studies done in awake, behaving animals. Testing the implications of our findings will require new experiments that combine neuronal recordings with local infusions of muscarinic drugs and KCNQ channel blockers as well as optogenetic and chemogenetic manipulations of cholinergic activity (e.g., ChAT rats) in the mPFC.

### Potential Neuronal Circuits for the Regulation of Palatability

The mPFC region examined in the present study is one part of a large brain network that encodes the value of foods and regulates consummatory behavior. We have emphasized a role of the mPFC in the *expression* of palatability. There are neurons in the mPFC that are modulated by sensory (taste) properties of foods (Jezzini et al., 2013). However, a more likely candidate for encoding taste information or retrieving values determined by taste information from memory is the agranular insular cortex (AIC), which is classically considered as “taste cortex” (Yamamoto et al., 1989). The AIC contains neurons that encode for the palatability of tastants (Grossman et al., 2008) and respond more vigorously and with shorter latencies to specific tastants compared to the mPFC (Jezzini et al., 2013). The source of these palatability signals within the AIC may be the basolateral amygdala (Grossman et al., 2008) which projects to both the AIC and the mPFC (Hoover and Vertes, 2007; Reppucci and Petrovich, 2015).

Several studies have implicated the AIC in the reward-guided control of action (DeCoteau et al., 1997; Ragozzino and Kesner, 1999; Balleine and Dickinson, 2000; Kesner and Gilbert, 2007; Gardner and Fontanini, 2014; Kusumoto-Yoshida et al., 2015), but not other mPFC dependent behaviors such as action timing (Smith et al., 2010) and delayed alternation (Horst and Laubach, 2009). This region seems to be involved in the retrieval of outcome values that are encoded by the basolateral amygdala (BLA; Parkes and Balleine, 2013). However, as reversible inactivations of the AIC and mPFC have comparable effects on palatability driven feeding (Baldo et al., 2015), we propose that the two areas work together with BLA to regulate consummatory behavior, by enabling the conversion of reward values into control signals that guide action selection (e.g., lick now or later in the incentive contrast licking task).

Anatomical tract-tracing studies have reported heavy interconnections between the mPFC and AIC (Gabbott et al., 2003) and there are significant inputs from the BLA to the region of mPFC that was the focus of the present study (Bacon et al., 1996). Inputs from BLA terminate on parvalbumin interneurons in the mPFC (Gabbott et al., 2006), which regulate the dynamics of neuronal in the mPFC (Dilgen et al., 2013). These connections could both provide value signals to the mPFC and shape the timing of neuronal activity associated with the initiation of consummatory behavior, as described by Horst and Laubach (2013). The BLA also directly innervates corticospinal neurons in the mPFC (Gabbott et al., 2012), which are associated with the autonomic nervous system (Gabbott et al., 2005). Through these connections, information about the palatability of an ingested food or fluid may be processed in the mPFC and modulated by cholinergic tone and cerebrospinal levels of ghrelin to control consummatory behavior.

In addition to its corticospinal connections, the mPFC sends dense projections to autonomic and feeding-related centers in the hypothalamus (Floyd et al., 2001), midbrain (Floyd et al., 2000), and brainstem (Gabbott et al., 2005), including a recently described projection to a trigeminal relay in the brainstem (Iida et al., 2010). The target of the mPFC in the lateral hypothalamus has recently been shown to contain neurons that encode palatability-related information (Li et al., 2013) and to become phasically active in relation to licking behavior (Tandon et al., 2012). Another major output of the mPFC is the ventral striatum, a region associated with encoding reward values (Bissonette et al., 2013) and controlling food seeking behaviors (Taha and Fields, 2005). Cholinergic or ghrelinergic modulation of any of these projections, acting through KCNQ channels, could influence neuronal activity in these subcortical centers to regulate the expression of palatability. This neuronal circuit interpretation of our findings could be tested in new studies that involve multi-site neuronal recordings and opto-/chemo-genetic perturbations of neuronal recordings at the specific times when animals initiate consummatory actions.

### Prefrontal vs. Hypothalamic Effects of Ghrelin

A novel finding of the present study is that the direct administration of ghrelin into the mPFC alters a specific behavioral measure of palatability (i.e., the duration of licking bouts). This finding is in contrast to a recent study in which ghrelin was infused into the ventricles near the ventral hypothalamus (Overduin et al., 2012). The Overduin study found that ghrelin increases overall intake but does not increase measures of palatability. This difference between these findings is likely due to actions of ghrelin on different brain areas (hypothalamus vs. mPFC). Feeding centers in the hypothalamus contain neurons such as the agouti-related pepride-secreting (AgRP) neurons that are sensitive to ghrelin but do not influence palatability (Denis et al., 2015). Our finding that ghrelin is able to influence the expression of palatability may simply be due to ghrelin acting on the same ion channels that the muscarinic cholinergic system acts on (KCNQ channels) and the subsequent modulation of consummatory related neuronal activity in the mPFC (i.e., increases in firing and increased gain of transmission in the licking (theta) frequency). This interpretation of our results could be tested in future studies that combine neuronal recordings with local drug infusions or opto-/chemo-genetic manipulations of neurons with ghrelin receptors in the mPFC and hypothalamus.

## Funding

Financial Support: National Science Foundation grant 1121147, National Institutes of Health grant DK099792-01A1, and two grants from the Klarman Family Foundation to ml.

## Conflict of Interest Statement

The authors declare that the research was conducted in the absence of any commercial or financial relationships that could be construed as a potential conflict of interest.

## Footnotes

^1^http://www.r-project.org
